# Redo-TAVR: Essential Concepts, Updated Data and Current Gaps in Evidence

**DOI:** 10.3390/jcm12144788

**Published:** 2023-07-20

**Authors:** Attílio Galhardo, Marisa Avvedimento, Siddhartha Mengi, Josep Rodés-Cabau

**Affiliations:** Quebec Heart & Lung Institute, Laval University, Quebec City, QC G1V 4G5, Canada; attiliogalhardo@hotmail.com (A.G.); marisa.avvedimento.1@ulaval.ca (M.A.); siddhartha.mengi.1@ulaval.ca (S.M.)

**Keywords:** redo-TAVR, ViV-TAVR, bioprosthetic valve degeneration, coronary obstruction, coronary access

## Abstract

Within the last two decades, transcatheter aortic valve replacement (TAVR) has transformed the treatment strategy for symptomatic severe aortic stenosis (AS), representing a less invasive alternative to traditional open-chest surgery. With time, advances in device features, imaging planning, and implantation techniques have contributed to an improvement in safety as well as a reduction in procedural complications. This has led to the expansion of TAVR to lower-risk patients, where TAVR has shown favorable outcomes compared to surgical aortic valve replacement (SAVR). As TAVR expands to younger and lower-risk patients with longer life expectancies, the need for reintervention for failing transcatheter heart valves is expected to increase. Redo-TAVR has gained increasing relevance in the lifetime management of AS as one of the treatment strategies available for structural valve dysfunction (SVD). However, some issues are associated with this approach, including coronary re-access and the risk of coronary obstruction. In this review, we provide essential concepts to properly select candidates for Redo-TAVR, updated data on clinical outcomes and complication rates, and current gaps in evidence.

## 1. Introduction

Originally described by Cribier in 2002 [1], transcatheter aortic valve replacement (TAVR) has since proven to be a safe, effective solution for replacing the aortic valve throughout the full spectrum of surgical risk patients with severe aortic stenosis [2,3,4]. Current data show that since its approval by the Food and Drug Administration (FDA) as an alternative to SAVR for high (2012), moderate (2016), and low (2019) surgical risk patients, TAVR has been established as a therapy with high adherence by physicians and patients, outpacing the volume of all forms of SAVR [5].

As TAVR expands to younger and lower risk patients with longer life expectancies, the need for reintervention for failing transcatheter heart valves (THV) is expected to increase. Over time, several efforts have been made to mitigate procedural risks, with the aim of maximizing its durability. Currently, Redo-TAVR provides acceptable results in terms of safety and efficacy, but it does come with some unavoidable procedural risks that cannot be neglected [6].

In this review, we present essential concepts for selecting appropriate candidates for Redo-TAVR, provide updated data on outcomes and complication rates, and discuss current gaps in evidence.

## 2. Bioprosthetic Valve Dysfunction and Structural Valve Deterioration Definitions

**Figure 1:**
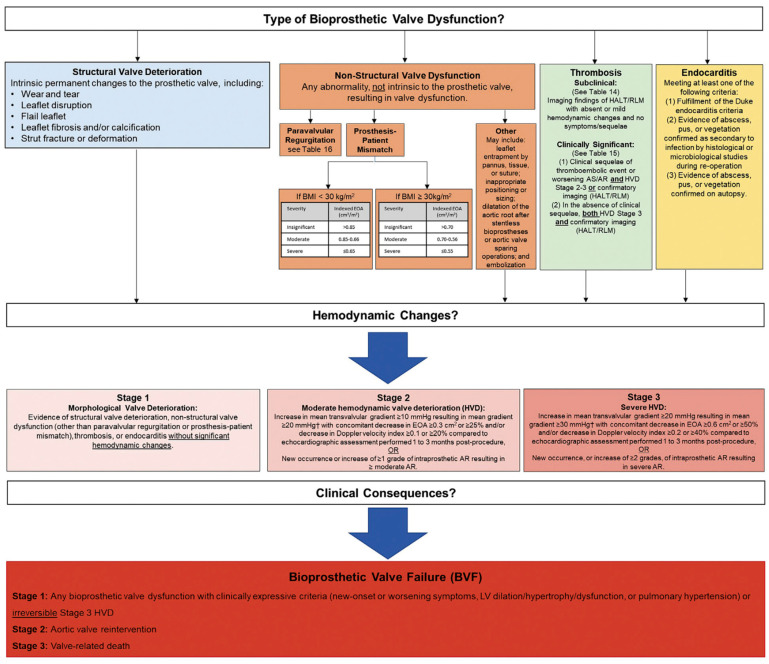
Bioprosthetic Valve Dysfunction and Bioprosthetic Valve Failure definitions according to VARC-3 criteria. *From Valve Academic Research Consortium 3: Updated Endpoint Definitions for Aortic Valve Clinical Research. J. Am. Coll. Cardiol. 2020, 76, 2492–2516* [7]. Permission to reprint obtained.

The Valve Academic Research Consortium (VARC) published its most recent updates in response to the exponential growth in the number of transcatheter and surgical aortic valve interventions in order to standardize clinical endpoints for both procedures [7]. Bioprosthetic Valve Dysfunction (BVD) and Structural Valve Deterioration (SVD) were included as novel endpoints in the VARC-3 document (Figure 1). BVD was subcategorized into four groups: structural valve deterioration (the dysfunction is related to an intrinsic permanent change in the prosthetic valve, such as wear and tear, leaflet disruption, flail leaflet, leaflet fibrosis and/or calcification and strut fracture), non-structural valve dysfunction (such as paravalvular regurgitation, prosthesis-patient mismatch, leaflet entrapment by pannus, tissue or suture, inappropriate positioning, or sizing), thrombosis, and endocarditis. Three stages of BVD were proposed according to hemodynamic changes: Stage 1: morphological valve deterioration, where no hemodynamic change is present; Stage 2: moderate hemodynamic deterioration; and Stage 3: severe hemodynamic valve deterioration.

The definition of bioprosthetic valve failure (BVF) included in this document states that this is a patient-oriented clinical endpoint that considers relevant and clinically meaningful consequences of BVD, including stage 3 hemodynamic valve degeneration and irreversible changes in hemodynamics as a result of SVD, as well as clinical symptoms and sequelae, including valve-related death and re-intervention (either surgical or transcatheter).

## 3. TAVR Explantation

Prosthesis failure mechanism, patient’s anatomical characteristics, age, comorbidities, and life expectancy are among the factors that should be considered when deciding what is the best alternative for a failed transcatheter bioprosthetic valve.

TAVR explantation consists of surgically removing a transcatheter aortic valve device from the patient’s body. It requires a skilled surgical team and is associated with an increased risk of complications. Nevertheless, for selected patients, this may be the strategy of choice as a second intervention. Data from the multicenter, international EXPLANT-TAVR registry reported elevated mortality and stroke rates regardless of the procedure’s timing and primary indication for TAVR explantation [8]. According to that study, after one year, the overall mortality rate was 28.5% and the stroke rate was 18.7%. Up to 63% of patients undergoing THV explantation needed concomitant procedures, such as aortic repair, mitral or tricuspid interventions, or coronary artery bypass grafting [9,10].

In terms of device type, 30-day mortality and need for any concomitant cardiac procedures at the time of TAVR explant were comparable between self- (SEV) and balloon-expandable (BEV) valves, with a higher rate of ascending aortic repair related to self-expandable devices (22% vs. 9%; *p* < 0.001) [9].

Newly released results from the EXPLANTORREDO-TAVR registry, including 542 patients from 29 centers who underwent TAVR reintervention (TAVR-explant or Redo-TAVR) due to THV failure, suggested that neither the mechanism of degeneration (SVD or non-SVD) nor the device design (BEV or SEV) significantly impacted mortality after TAVR reintervention [11].

As for outcomes of TAVR explant versus aortic root replacement after failed TAVR, overall survival at a mean follow-up of 6.9 months was 81.2%, with no differences in in-hospital, 30-day, and 1-year stroke and mortality rates between the two groups [12].

## 4. Overall Mortality

A recent meta-analysis including 16,207 patients, showed lower 30-day mortality rates for valve in valve (ViV) TAVR compared to redo-SAVR (OR: 0.52; 95% CI: 0.39 to 0.68; *p* < 0.001), with no differences regarding 1-year mortality, stroke, permanent pacemaker implantation, myocardial infarction, acute kidney injury requiring dialysis, major vascular complications, and paravalvular leak [13]. Consistently, TAVR-in-TAVR procedures showed low in-hospital mortality (1.25%) with a success rate of 86.8% [14].

In a large multicentric analysis performed by Landes et al., Redo-TAVR emerged as a safe and effective option for THV late failure, with a mortality of 1.5% and 11.7% at 30 days and 1 year, respectively [15]. Redo-TAVR was associated with a high device success (85.1%) and acceptable peri-procedural complication rates (stroke: 1.4%, coronary obstruction: 0.9%, and new permanent pacemaker: 9.6%) [15]. This percentage of coronary obstruction was even lower than the one reported for TAVR-in-SAVR in the VIVID registry (2.3%) [16].

Although a possible patient selection bias cannot be excluded, these data put Redo-TAVR as a safe and effective option for THV failure, with mortality rates of 1.5% and 11.7% at 30 days and 1 year, respectively [15].

More recently, a study using a propensity score matching analysis compared TAVR-in-TAVR versus TAVR-in-SAVR. Endpoints favored TAVR-in-TAVR in terms of procedural success and showed similarities between the two strategies regarding safety and mortality [17]. However, an important caveat was that the time interval between the first and second procedures was excluded from the propensity score matching analysis (median of 3 years in TAVR-in-TAVR versus 9 years in TAVR-in-SAVR).

## 5. Preprocedural Computed Tomography Planning

**Figure 2:**
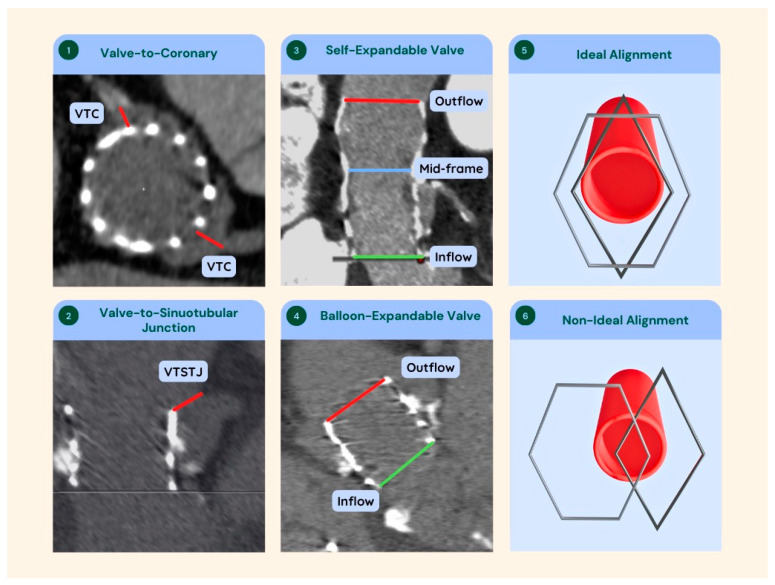
Tomographic Measurements and Importance of Coronary-TVH Alignment in TAVR-in-TAVR. (**1**,**2**) Tomographic images exemplifying the VTC (**1**) and VTSTJ (**2**) measurement sites. (**3**,**4**) Longitudinal sections of BEV (**3**) and SEV (**4**) indicating inflow, mid-frame, and outflow diameters. (**5**,**6**) Three-dimensional representation in perspective between the coronary ostium and two layers of stent cells in a situation of ideal (**5**) and non-ideal (**6**) alignment between the three planes.

In order to properly plan a redo-TAVR procedure, it is imperative to collect information about the prosthesis implanted at the index procedure, such as valve type and size, neoskirt height, sinotubular junction (STJ) diameter, and coronary height (Figure 2). 

Identifying the posts of the first prosthesis and considering coronary safety techniques are essential to reduce the risk of coronary complications. In addition, valve-to-sinotubular junction (VTSTJ) measurements smaller than 2 mm make re-access with 6F catheters unfeasible, which leads to sinus sequestration.

When analyzing a tomography with a prosthesis in the aortic position, it is important to consider possible artifacts. Motion artifacts can completely derail the analysis and lead to wrong decisions. Metal structures and significant calcifications may give the impression that the objects are larger than their true size, leading to an underestimation of the true inner diameter (ID) and valve-to-coronary (VTC)/VTSTJ measurements. This is called “blooming artifacts”. To minimize its interference in measurements it is crucial to reduce image gain and position the markers in the center of the structure [18]. 

Important steps to properly evaluate the computed tomography (CT) scan prior to the Redo-TAVR procedures are summarized in Table 1.

## 6. TAVR-in-TAVR: Procedural Complications

### Coronary Obstruction

Coronary artery disease is one of the most frequent comorbidities among TAVR candidates and the optimal management in this population still lacks definitive data [19]. A few scenarios are supported by current guidelines for percutaneous coronary intervention (PCI) in patients undergoing TAVR; however, the level of evidence is limited, and the recommendation is only moderately strong [20]. Meanwhile, recent evidence showed no benefit in short- or mid-term mortality with routine PCI pre-TAVR, with a trend towards an increased risk for bleeding events [21,22]. 

**Figure 3:**
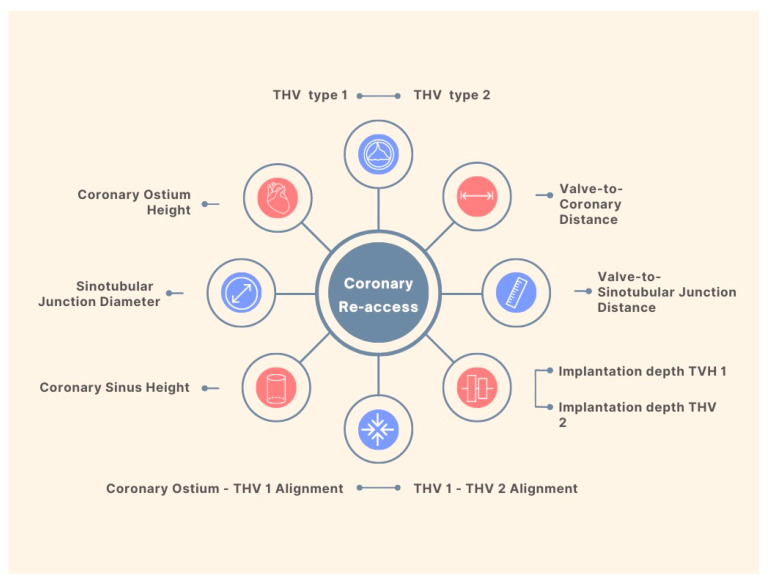
Factors Influencing Coronary Re-Access Following Redo-TAVR.

Data from a multicenter registry indicate a rate of 0.9% of unplanned percutaneous coronary revascularization after TAVR [23]. This number is expected to increase given the implantation of THV in patients with lower surgical risk, and a relatively younger population. Although registries report high success rates of coronary cannulation in patients with acute coronary syndromes and THVs [24], the feasibility of coronary access after a Redo-TAVR remains a big concern. Factors impacting coronary re-access after Redo-TAVR procedures are summarized in Figure 3.

Researchers compiled CT scans from patients who had undergone TAVR and the findings indicated that, among those with CoreValve/Evolut and Sapien valves, 90% and 67% respectively had coronary arteries positioned below the top of the neoskirt. The risk for technically impossible coronary access was 27% and 10% in CoreValve/Evolut-first and SAPIEN-first cases, respectively [25].

Detailed analysis of the first TAVR is of utmost importance in planning the second procedure. Knowing the patient’s anatomical characteristics and device type allows the assistant team to anticipate potential challenges such as sinus of Valsalva sequestration and coronary obstruction.

A recent benchtop study evaluated 38 combinations of 5 valve designs (Sapien3, Evolut Pro, Acurate Neo, and Portico in Sapien XT or Evolut R) in order to evaluate neoskirt and implant heights, and their influence on coronary re-access. Taller implants were associated with larger neoskirts in THVs such as Evolut R and Acurate, with the Evolut-in-Evolut combination responsible for the highest value (31.6 mm) [26]. 

The choice of the second valve (supra or intra-annular design, balloon or self-expanding) is certainly a critical point in Redo-TAVR. To date, there are no formal guidelines that indicate or contraindicate combinations of devices. Anatomical and post-first TAVR characteristics must be combined with the patient’s profile, the expertise of the medical team, and the resources available at the center. 

Although TAVR is proven to be an effective treatment in bicuspid aortic valve disease, there are limited data on Redo-TAVR in these patients [27,28]. Based on CT scans, a study found that the risk of coronary obstruction during Redo-TAVR was comparable between tricuspid and bicuspid type 1 aortic valves, while it was significantly lower in type 0 bicuspid aortic valves [29].

**Figure 4:**
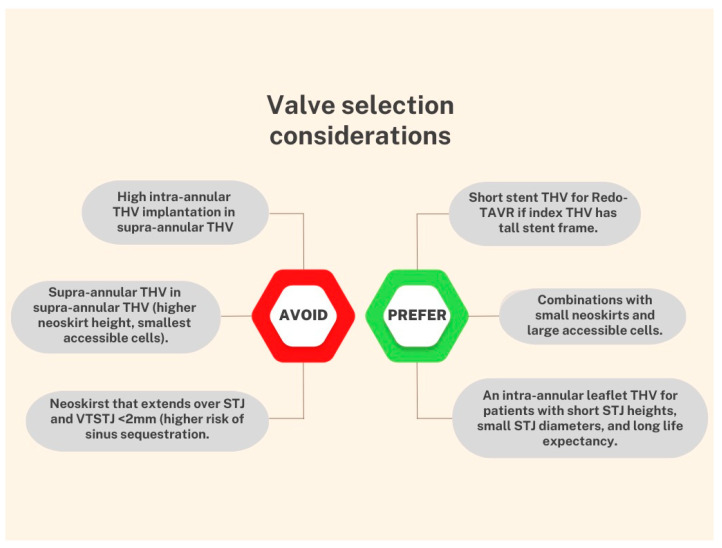
Considerations for choosing a device combination.

The local heart team plays a crucial role in the decision-making process, and it is essential to anticipate potential complications in advance. The selection of the valve and the associated procedural strategy must be tailored on a case-by-case basis. Based on the available data, some suggestions for device combinations are shown in Figure 4.

## 7. Mitigating the Risk of Coronary Obstruction

If the patient is deemed to be at a high risk of coronary obstruction after CT analysis, some techniques may be performed to intentionally lacerate the leaflet that may end up in front of the coronary ostia. First described in 2018, the BASILICA (Bioprosthetic or native Aortic Scallop Intentional Laceration to prevent Coronary Artery obstruction) technique has been used with considerable success rates [30,31,32]. However, there are some scenarios where laceration may not be wide enough, such as small VTC distance, TAVR-in-TAVR, and leaflets with bulky calcifications due to suboptimal leaflet “splay”. In these cases, the balloon-assisted BASILICA can be an alternative. This technique consists of inflating a non-compliant balloon into the leaflet before the conventional laceration, allowing a more extensive cross-sectional area and wider leaflet splay [33].

Dedicated transcatheter leaflet-splitting devices are another promising tool for patients with threatened coronary obstruction. Newly published data from the Shortcut device have proven its safety and effectiveness for leaflet modification. This new device uses a blade to make a mechanical laceration and simplifies the many steps of traditional electrosurgery techniques [34]. 

Notably, leaflet modification techniques will only be effective in patients whose prosthesis posts are not in front of the coronary ostium. Therefore, commissural alignment is of utmost importance to allow such techniques to be successfully performed and provide a significant benefit to patients who are unable to undergo open-chest procedures [35]. 

A disadvantage of current leaflet modification techniques is the lack of control over the final leaflet position, which occurs randomly. Depending on its position during the expansion of the second prosthesis, the splay leaflet may assume a suboptimal opening and compromise the coronary flow. Another pitfall that can jeopardize an excellent result after leaflet laceration is the new position of the TAVR device skirt. High positions can obstruct the opening caused in the leaflets. 

## 8. Patient-Prosthesis Mismatch

Patient-prosthesis mismatch (PPM) in Redo-TAVR can result from a variety of factors, including changes in the anatomy of the patient’s aortic root or changes in the size of the patient’s previous artificial valve. 

Diagnosing and managing PPM in Redo-TAVR patients may be important for optimizing their outcomes [36]. Prevention strategies can include further imaging studies, valve re-sizing, or even re-intervention.

According to the TRANSIT-PPM registry, the prevalence of severe and moderate PPM after Redo-TAVR is 6.5% and 14.2%, respectively. The study revealed that the incidence of severe PPM was notably higher among patients who received a supra-annular SEV THV as a second prosthesis. Specifically, the rate of severe PPM was significantly higher among cases of a SE TAVR implanted into a balloon-expandable device [37].

As for ViV-TAVR, a recently published systematic review including 3339 patients from 23 studies highlighted that moderate–severe PPM ranged from 11% to 58% regardless of the surgical valve type addressed [38].

When comparing the two strategies, it becomes apparent that there is a significant variation in the rates of PPM after ViV-TAVR, which tends to be higher than the rates observed after Redo-TAVR. 

### 8.1. Stroke

Despite refinements in device technology and increased operator experience, stroke is still a feared complication in patients undergoing TAVR and is associated with a significant increase in mortality risk [39]. In Redo-TAVR procedures, the reported periprocedural stroke rate ranges between 1.4% [15] and 1.8% [40], which is comparable with the stroke rates reported in randomized clinical trials in low-risk patients [4,41]. In clinical practice, no data are supporting the widespread adoption of cerebral embolic protection devices. As demonstrated in the PROTECTED-TAVR trial, strokes within 72h and disabling stroke rates were numerically lower in the group that used the SENTINEL device. However, statistical significance could not be established [42].

More evidence is needed to identify specific subgroups of patients undergoing Redo-TAVR procedures who may benefit more from the use of embolic protection devices.

### 8.2. Permanent Pacemaker Implantation

Conduction disturbances are still the most frequent periprocedural complication of TAVR despite major procedural improvements in recent years [43]. Baseline right bundle branch block, and valve implantation depth are associated with a higher risk of permanent pacemaker implantation (PPMI) after the index intervention [44].

Data from VIVID registry showed that for patients undergoing ViV-TAVR, the current generation of prosthetic valve systems are associated with a low incidence of PPMI (6.4%), especially with the use of new (vs. old) generation THVs (4.7% vs. 7.4%; *p* < 0.017) [45]. These data are consistent with the other two series reporting a PPI rate of 9.6% [44] and 8.7% after TAVR-in-TAVR [15].

After Redo-TAVR, the decision to implant a permanent pacemaker should be carefully evaluated and based on a careful evaluation of the patient’s risk factors, as well as the duration and degree of conduction disturbances that have occurred. To detect and intervene if any conduction abnormalities occur following Redo-TAVR, closer follow-up is crucial.

## 9. Post-Procedural Antithrombotic Management

The use of materials of animal origin, synthetics as well as metals in prosthetic valves confers higher rates of thromboembolic events in patients undergoing these therapies. In addition to the often-present atherosclerosis and atrial fibrillation, other factors that may contribute to this include aortic debris, calcium dislodgement, xenograft-related hemocompatibility, and increased platelet reactivity [46,47]. 

The optimal anti-thrombotic strategy after ViV procedures remains a subject of ongoing research and debate in the medical community. Currently, there is no consensus on the best approach, and the choice of anti-thrombotic therapy is often individualized based on multiple factors such as patient age, comorbidities, and the type of procedure performed.

Important documents offer guidance on antithrombotic management following a first TAVR [48,49]. However, for Redo-TAVR, no data are available to guide the antithrombotic management in this population, meaning that the ideal antithrombotic strategy remains to be determined. 

It is reasonable to assume that the presence of a second prosthesis and the neoskirt may increase thrombotic risk. This potential increase could prompt consideration of using anticoagulation treatment in these patients.. However, studies on anticoagulation therapy after TAVR in patients with no formal indication for anticoagulation have been negative [50]. Future studies may provide more data on this topic. Until further evidence is available, anti-thrombotic management after Redo-TAVR should be discussed case-by-case, based on the patient’s thrombotic and bleeding risk.

## 10. Future Perspectives

The topic of Redo-TAVR is a growing field that has yet to be fully explored. Since the number of repeated TAVR cases is increasing every year, evidence for the topic is becoming less scarce. 

Notably, only Edwards Lifesciences THV has received approval from the FDA for TAVR-in-TAVR procedures. However, it is expected that, in the coming years, other companies will also look after having their aortic platforms certified by regulatory agencies, empowering physicians with a broader range of options to choose from.

In addition to coronary re-access, future studies should also shed light on whether optimal commissural alignment contributes to improving valve hemodynamics and durability.

Studies on the impact of prosthesis implantation depth have demonstrated that a higher prosthesis implantation position results in lower conduction disturbances and PPMI rates. This strategy, however, can result in more difficult coronary access if a Redo-TAVR is eventually performed.

It is generally not recommended to change the optimal implant position of the first prosthesis while considering future interventions. However, for a small group of patients who are already dependent on a pacemaker before the first TAVR, and whose life expectancy outlives the estimated durability of the prosthesis, a “not-so-high” position for the first prosthesis might be considered.

Several studies have shown that leaflet modification techniques are both feasible and effective. These practices can be considered when dealing with patients at a high risk of coronary obstruction during the intervention. However, there is still a need for improvements to minimize the probability of the torn leaflet being randomly placed in an unfavorable position in the future.

To date, two ongoing 2 trials are including Redo-TAVR as a strategy for failing transcatheter bioprosthetic valves. (PARTNER 3 Trial—Aortic Valve-in-Valve— NCT03003299; ReTAVI prospective observational registry – NCT05601453).

## 11. Conclusions

**Figure 5:**
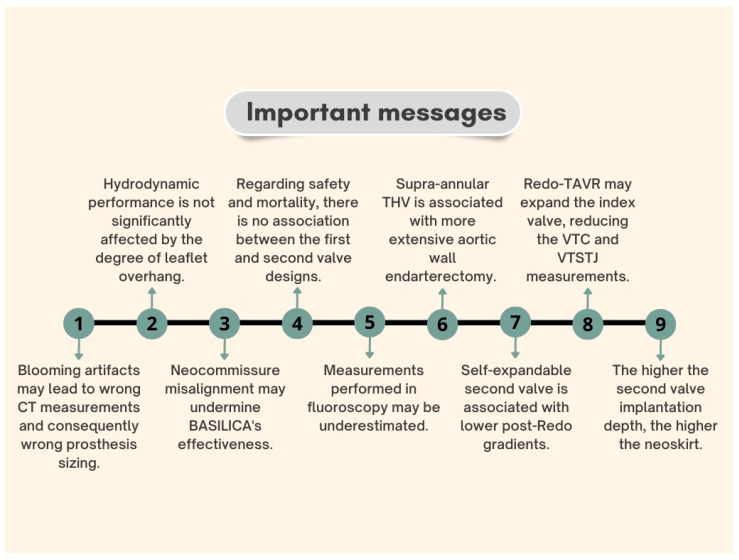
Redo-TAVR planning and execution concepts.

The consolidation of TAVR for all the spectrum of surgical risk patients is undeniably a paradigm shift in treating severe aortic stenosis. The achievement of this milestone brings with it the responsibility of providing safe and effective solutions for patients facing the inevitable degeneration of their first THV. Figure 5 presents some fundamental concepts for good planning and execution of Redo-TAVR.

**Figure 6:**
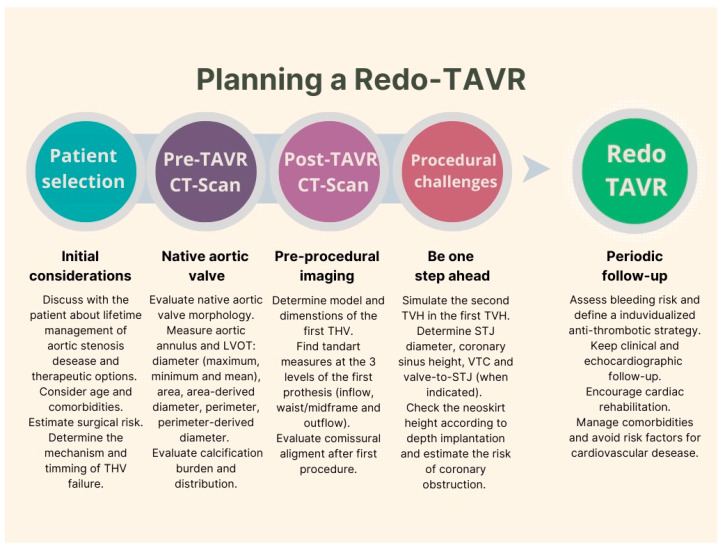
Redo-TAVR planning: essential points to consider.

The key to a successful Redo-TAVR begins with the index procedure or even earlier. This creates the need to better understand and refine pre-procedural optimization strategies necessary to achieve optimal outcomes with the first implantation. A summary of the essential points to consider when planning a Redo-TAVR is shown in Figure 6.

Despite the growing number of publications regarding Redo-TAVR, evidence still lacks robustness. Current evidence supports considering TAVR-in-TAVR as a promising alternative to TAVR explantation. However, it is still pertinent to assess the risks associated with Redo-TAVR. Furthermore, there is a lack of long-term data in this field. 

It may be premature to interpret current data on Redo-TAVR as representative of real-world practice, but the procedure is demonstrating promising potential for selecting patients and yielding acceptable outcomes. It is also advisable to add a note of caution when comparing Redo-TAVR outcomes related to the device design of the failed THV.

Due to the limited number of studies and the evolving designs of the leading valve platforms on the market, recommendations regarding device selection for Redo-TAVR currently lack robust evidence. However, some relevant theoretical points can be utilized to predict potential challenges or complexities.

## Figures and Tables

**Table 1:** Recommendation for analyzing CT scans before Redo-TAVR.

Analyze native valve images looking for calcification and its distribution, coronary height, bulky calcified leaflets, dilated left ventricular outflow tract (LVOT), and calcified STJ.
Before starting the measurements, check for artifacts and adjust image gain, if necessary.
Measure the inflow diameter, the middle part, and the outflow diameter of the first prosthesis. Compare these dimensions to the native valve and decide whether the first prosthesis expansion can be optimized.
Estimate the size of the neoskirt that will be formed by knowing the height of the leaflets of the first prosthesis and the implantation depth of the second valve.
Evaluate the alignment between the coronary ostium and posts of the first prosthesis to predict whether leaflet modification techniques are feasible.
Evaluate the height of the coronary ostia in relation to the height of the first valve stent.
If the stent is framed below the coronary ostium, measure VTC. If the stent frame is above the coronary ostium, measure VTSTJ.

## Data Availability

All data underlying this article will be shared on reasonable request to the corresponding author.

## Abbreviations

AS	aortic stenosis
BEV	balloon-expandable valve
BVD	bioprosthetic valve dysfunction
BVF	bioprosthetic valve failure
CT	computed tomography
EOA	effective orifice area
LVOT	left ventricular outflow tract
OR	odds-ratio
PCI	percutaneous coronary intervention
PPMI	permanent pacemaker implantation
PPM	patient-prosthesis mismatch
SAVR	surgical aortic valve replacement
SEV	self-expandable valve
STJ	sinotubular junction
SVD	structural valve degeneration
TAVR	transcatheter aortic valve replacement
THV	transcatheter heart valve
VARC	Valve Academic Research Consortium
ViV	valve-in-valve
VTC	valve-to-coronary
VTSTJ	valve-to-sinotubular junction
